# In Vitro Human Skin Penetration, Antioxidant and Antimicrobial Activity of Ethanol-Water Extract of Fireweed (*Epilobium angustifolium* L.)

**DOI:** 10.3390/molecules26020329

**Published:** 2021-01-10

**Authors:** Anna Nowak, Krystyna Cybulska, Edyta Makuch, Łukasz Kucharski, Monika Różewicka-Czabańska, Piotr Prowans, Norbert Czapla, Piotr Bargiel, Jan Petriczko, Adam Klimowicz

**Affiliations:** 1 Department of Cosmetic and Pharmaceutical Chemistry, Pomeranian Medical University in Szczecin, PL-70111 Szczecin, Poland; lukasz.kucharski@pum.edu.pl (Ł.K.); adklim@pum.edu.pl (A.K.); 2 Department of Microbiology and Environmental Chemistry, Faculty of Environmental Management and Agriculture, West Pomeranian University of Technology, Szczecin, PL-71434 Szczecin, Poland; Krystyna.Cybulska@zut.edu.pl; 3 Department of Chemical Organic Technology and Polymeric Materials, Faculty of Chemical Technology and Engineering, West Pomeranian University of Technology, Szczecin, PL-70322 Szczecin, Poland; emakuch@zut.edu.pl; 4 Clinic of Skin and Venereal Diseases, Pomeranian Medical University in Szczecin, PL-72010 Police, Poland; monroz@pum.edu.pl; 5 Department of Plastic, Endocrine and General Surgery, Pomeranian Medical University in Szczecin, PL-72010 Police, Poland; piotr.prowans@pum.edu.pl (P.P.); norbert.czapla@pum.edu.pl (N.C.); piotr.bargiel@pum.edu.pl (P.B.); jan.petriczko@pum.edu.pl (J.P.)

**Keywords:** herbal extract, antibacterial activity, skin, Franz cell, phenolic acids, antioxidants

## Abstract

*Epilobium angustifolium* L. is applied as an antiseptic agent in the treatment of skin diseases. However, there is a lack of information on human skin penetration of active ingredients with antioxidative potential. It seems crucial because bacterial infections of skin and subcutaneous tissue are common and partly depend on oxidative stress. Therefore, we evaluated in vitro human skin penetration of fireweed ethanol-water extracts (FEEs) by determining antioxidant activity of these extracts before and after penetration study using 2,2-diphenyl-1-picrylhydrazyl (DPPH), 2,2′-azino-bis(3-ethylbenzothiazoline-6-sulfonic acid) (ABTS), and Folin–Ciocalteu methods. Microbiological tests of extracts were done. The qualitative and quantitative evaluation was performed using gas chromatography-mass spectrometry (GC-MS) and high-performance liquid chromatography (HPLC-UV) methods. The in vitro human skin penetration using the Franz diffusion chamber was assessed. The high antioxidant activity of FEEs was found. Gallic acid (GA), chlorogenic acid (ChA), 3,4-dihydroxybenzoic acid (3,4-DHB), 4-hydroxybenzoic acid (4-HB), and caffeic acid (CA) were identified in the extracts. The antibacterial activities were found against *Serratia lutea*, *S. marcescens*, *Bacillus subtilis*, *B. pseudomycoides*, and *B. thuringiensis* and next *Enterococcus faecalis*, *E. faecium*, *Streptococcus pneumoniae*, *Pseudomonas aeruginosa*, and *P. fluorescens* strains. In vitro penetration studies showed the penetration of some phenolic acids and their accumulation in the skin. Our results confirm the importance of skin penetration studies to guarantee the efficacy of formulations containing *E. angustifolium* extracts.

## 1. Introduction

Fireweed (*Epilobium angustifolium* (L.) Holub) (Onagraceaeis) is a well-known medicinal plant [1,2] due to its anti-inflammatory, antioxidant [3,4], antibacterial [5], analgesic, and anti-cancer [3] properties. Traditionally, the infusion of leaves of this plant could be beneficial for headache, cold, gastrointestinal disorders, and prostate problems [3]. It is also used topically as an antiseptic for wounds and various skin and mucous membrane diseases [1,6]. Its pharmacological activity is due to the content of several bioactive compounds such as phenolic acids (PhAs), including benzoic acid derivatives, e.g., GA, 3,4-DHB, 4-HB, and cinnamic acid derivatives, e.g., CA [7]. The phenolic acids and other antioxidants in *E. angustifolium* are considered to be valuable therapeutic ingredients with antioxidant and antimicrobial properties [8] in preparations applied to the skin [9]. However, there is no information on human skin penetration and their accumulation in the skin or possible penetration into deeper tissues. Frequently bacterial infections located in the skin and the underlying tissues depend on oxidative stress [10]. For example, *S. aureus* infection induces reactive oxygen species (ROS) in macrophages, neutrophils, and leukocytes, increases free radical production, and reduces the antioxidant response of these cells [11,12], while oxidative-stress-generated responding to this bacteria can damage the injured skin [13]. More ROS is released during inflammation, which protects the body against microorganisms [14]. In addition, human skin is one of the main routes for penetration bacteria colonizing various areas. *Enterococcus*, *Streptococcus*, *Serratia*, *Pseudomonas*, and *Bacillus* are frequently transmitted by this route [15]. Considering the increasing bacterial resistance, plants with high antioxidant and antimicrobial activity are increasingly used as ingredients of cosmetics and therapeutics [16,17,18,19]. In recent years a greater interest in “natural” products, perceived by patients as safer than products containing “synthetic” ingredients, has been observed. Moreover, there is growing interest to obtain novel, low-cost, highly effective, and safe preparations [20]. However, for the agents used in the treatment of skin disorders, several limitations such as low penetration have been observed. Therefore, the aim of the study was to assess the chemical composition and the antioxidant and antibacterial activity of fireweed ethanol-water extracts (FEEs) as a valuable source of bioactive substances with antioxidant and antimicrobial properties and to evaluate the in vitro human skin penetration of selected FEE compounds and their accumulation in the skin. Such a study will help to assess the extent to which the active substances in *E. angustifolium* can be useful to protect not only the skin surface and its deeper layers but also the surrounding tissues against oxidative stress and bacterial infection.

## 2. Results

### 2.1. Chemical Composition of the FEE and Its Antioxidant Activity

**Figure 1:**
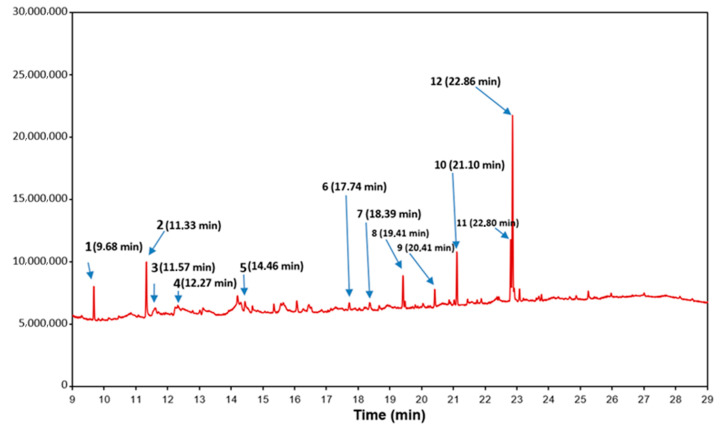
GC-MS chromatogram of the FEE.

Figure 1 presents the gas chromatography-mass spectrometry (GC-MS) chromatogram of the FEE. In Table 1 the qualitative and quantitative composition of the extract is summarized. The following groups of compounds were identified: oxygen derivatives of monoterpene hydrocarbons (compounds 1 and 5), unsaturated aliphatic alcohol (compound 2), camphene derivatives (compound 3), monocyclic unsaturated terpene ketones (compound 4), oxygen derivatives of sesquiterpene hydrocarbons (compounds 6 and 7), vitamin D derivative (compound 8), cyclic ether (compound 9), and fatty acid methyl esters (compounds 10, 11, and 12). Methyl esters of fatty acids seemed to be the significant components of the FEE, and the average percentage of oleic acid methyl esters was 15.2% (methyl palmitate), 9.6% (methyl linoleate), and 32.2% (methyl oleate). Other significant components of the analyzed extract were β-linalool (14.8%) and eucalyptol (10.3%), whereas oxygen derivatives of sesquiterpene hydrocarbons (compounds 6 and 7) constituted 2.4% of all identified compounds (Table 1).

### 2.2. Figures, Tables, and Schemes

**Figure 2:**
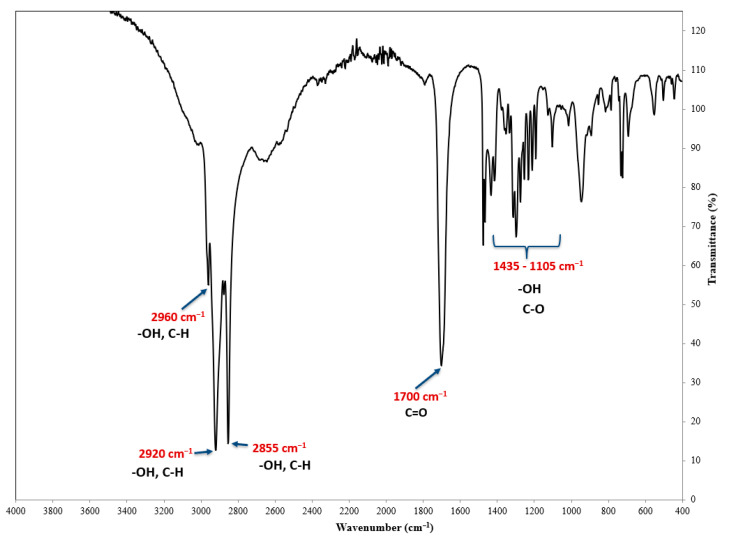
The IR spectrum of the FEE.

Figure 2 shows the IR spectrum of a sample containing the FEE. In the IR spectrum of the FEE, there is an absorption band at a wavenumber of about 1700 cm^−1^, which is characteristic to the carbonyl group, derived from ketones and esters. Carbonyl (ketone) groups can be derived from camphor, while ketone (ester) groups can be derived from α-terpinyl acetate. There are also bands at wavenumbers of around 2960, 2920, and 2855 cm^−1^, attributed to the hydroxyl group’s stretching vibration (O-H). These groups can be derived from the following compounds: β-linalool, α-terpineol, and 24,25-dihydroxycholecalciferol. The occurrence of the absorption bands at the wavenumber mentioned (i.e., around 2960, 2920, and 2855 cm^−1^) is also attributed to the stretching vibrations originating from the C-H carbon atoms. The IR spectrum also showed absorption bands in the range from 1435 to 1105 cm^−1^, derived from the single-molecule stretching bonds of eucalyptol, α-caryophyllene oxide, and β-caryophyllene oxide (Figure 2).

**Figure 3:**
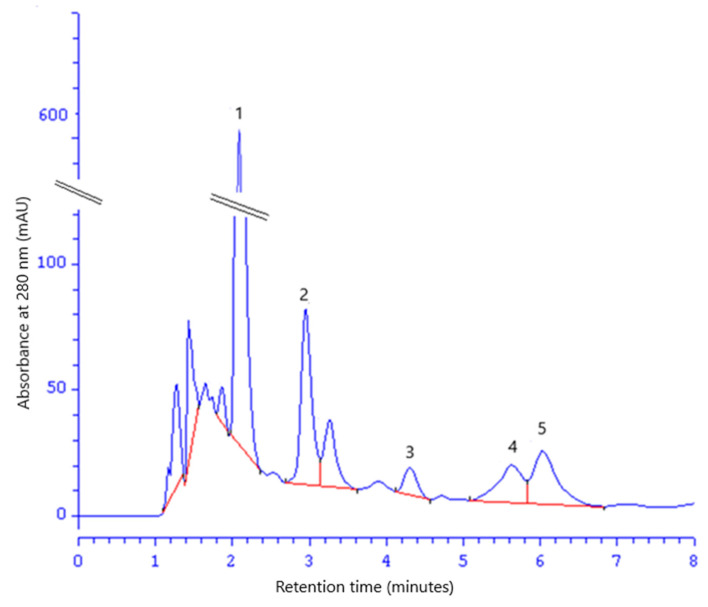
Chromatogram of phenolic acid identified in the FEE: gallic acid (1), 3,4-dihydroxybenzoic acid (2), 4-hydroxybenzoic acid (3), chlorogenic acid (4), and caffeic acid (5).

The HPLC method was used for the identification and quantification of selected phenolic acids in the FEE (Figure 3). The following phenolic acids were found: ChA, GA, 4-HB, 3,4-DHB, and CA. Their concentrations were GA 241.36 ± 4.25 mg/dm^3^, 3,4-DHB 165.19 ± 5.59 mg/dm^3^, 4-HB 118.16 ± 4.90 mg/dm^3^, ChA 64.35 ± 0.53 mg/dm^3^, and CA 54.29 ± 2.57 mg/dm^3^ (Table 2).

FEE was characterized by very high antioxidant activity, amounting with the DPPH method to 3.68 ± 0.02 mmol trolox/dm^3^ and 12.98 ± 0.04 mmol trolox/dm^3^ for ABTS, while the total polyphenol content determined by the Folin–Ciocalteu method was 1.94 ± 0.06 mmol GA/dm^3^ (Table 3).

### 2.3. Microbiological Assay

**Figure 4:**
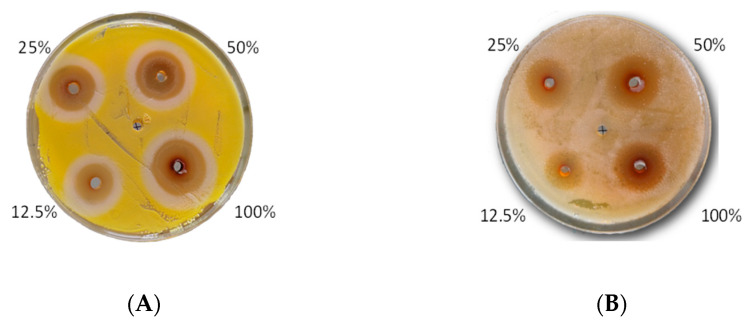
Photographs depicting the FEE reaction to limiting the growth of bacteria from genus *Serratia* sp. (**A**) and *Bacillus* sp. (**B**).

The analyzed extract showed antibacterial activity, but it depended on the analyzed strain (Table 4). The most sensitive strains of bacteria were from genus *Serratia* and from genus *Bacillus* (Figure 4). On the contrary, bacterial species from the genus *Enterococcus*, *Streptococcus*, and *Pseudomonas* were less sensitive. Here, the inhibition zone was about two times smaller than for the genus *Bacillus* and almost three times smaller than the genus *Serratia* (Table 4).

**Figure 5:**
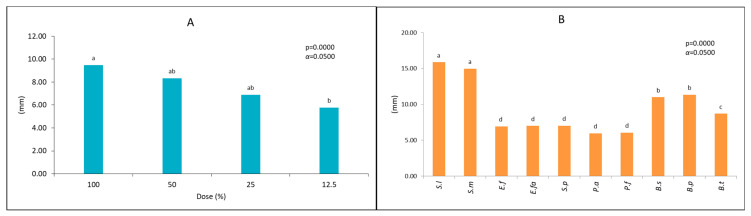
Mean effect of the different dose of the FEE on tested strains (**A**), mean susceptibility of the tested strains on the FEE (**B**). *S.l*: *Serratia lutea*; *S.m*: *Serratia marcescens*; *E.f*: *Enterococcus faecalis*; *E.f*: *Enterococcus faecium; S.p*: *Streptococcus pneumoniae*; *P.a*: *Pseudomonas aeruginosa*; *P.f*: *Pseudomonas fluorescens*; *B.s*: *Bacillus subtilis*; *B.p*: *Bacillus pseudomycoides*; *B.t*: *Bacillus thuringiensis.* Different letters: values differ significantly between analyzed samples.

**Figure 6:**
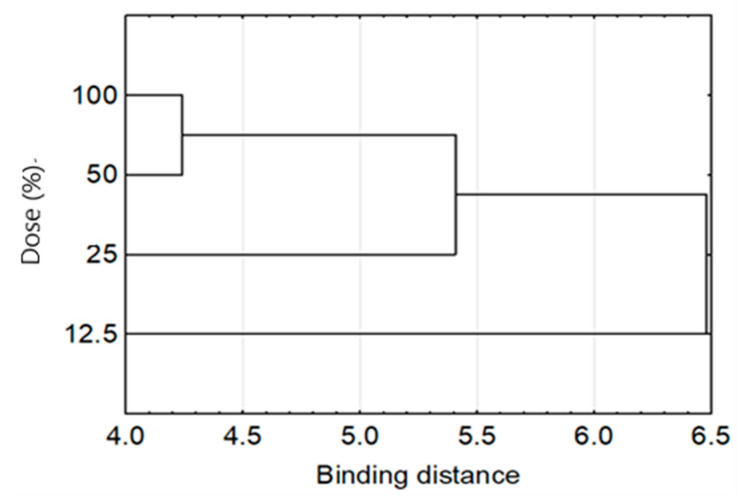
Cluster analysis graph for mean antimicrobial activity of the FEE.

In the study, four doses of the extract, i.e., 12.5%, 25%, 50%, and 100%, were used. In the case of *Enterococcus*, *Streptococcus*, and *Pseudomonas* strains, a smaller dependence of the bacterial reaction toward the extract dose’s size was observed (Table 4). The tested strains’ bacterial activity regularly decreased with the extract’s decreasing dose (Figure 5). A very similar effect of the two highest doses of the extract (100% and 50%) was found, and a different effect of the lowest dose (25% and 12.5%) (Figure 6).

### 2.4. Skin Penetration

Antioxidant activity and total polyphenol content were evaluated in the samples obtained during the in vitro human skin penetration study. The determinations were performed in plant extracts applied to the skin, in acceptor fluid collected after 24-h penetration, and in the fluid obtained after skin extraction following penetration completion. All the tested samples showed antioxidant activity, evaluated by the DPPH and ABTS methods. The acceptor fluid collected after the penetration test was completed, showing antioxidant activity of about 0.216 ± 0.08 mmol trolox/dm^3^ for the DPPH method and 0.519 ± 0.11 mmol trolox/dm^3^ for the ABTS method. Samples obtained after skin extraction following 24-h penetration were characterized by higher antioxidant activity: 0.456 ± 0.034 and 1.622 ± 0.57 mmol trolox/dm^3^ for DPPH and ABTS methods, respectively. A similar tendency for the total polyphenol content evaluated with the Folin–Ciocalteu method was observed. A higher content was found in the fluid obtained after skin extraction: 1.11 ± 0.11 mmol GA/dm^3^ as compared to the acceptor fluid collected after penetration: 0.59 ± 0.15 mmol GA/dm^3^ (Table 5).

**Figure 7:**
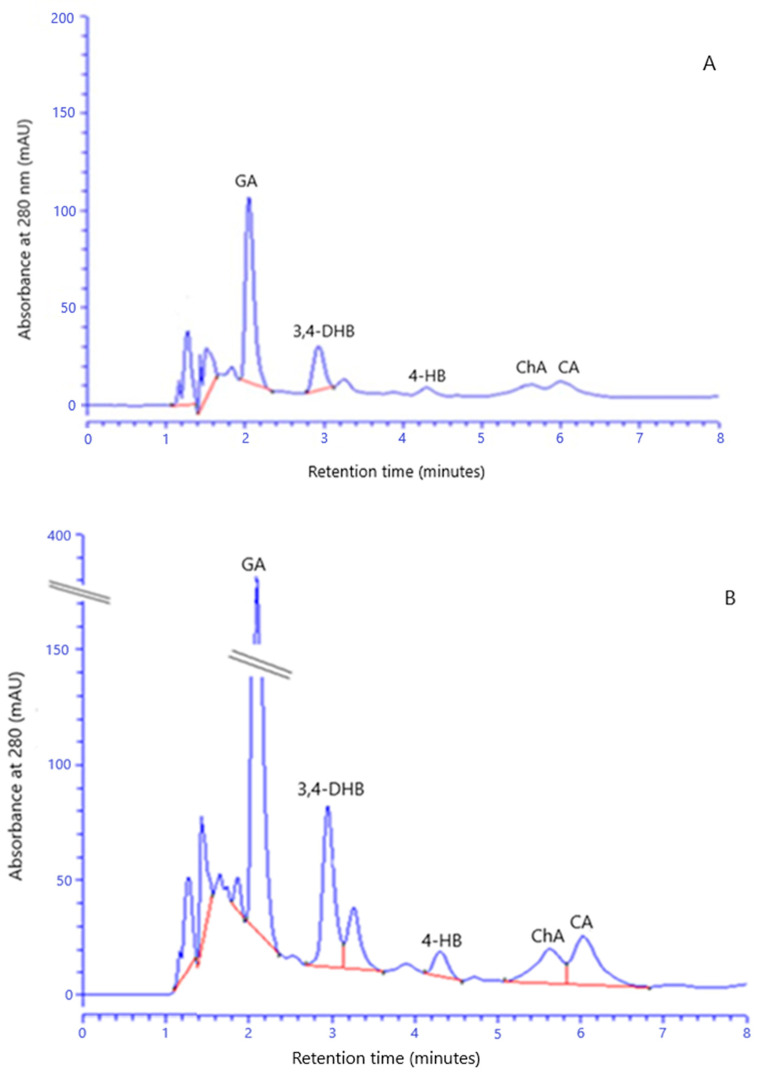
The HPLC chromatogram of acceptor fluid (**A**) and fluid after skin extraction (**B**), after 24-h penetration of the FEE.

Table 6 summarizes the content of selected phenolic acids in the acceptor fluid collected after 24-h penetration and in the skin collected after the end of the penetration of the applied FEE. Figure 7 shows the HPLC chromatogram of the acceptor fluid after 24-h penetration (7A) and the fluid recovered after skin extraction (7B).

From among the studied phenolic acids, GA, 3,4-DHB, and ChA penetrated to a higher degree than others; cumulative amounts of these acids penetrated during the 24-h study were 80.51 ± 8.27, 31.93 ± 1.12, and 30.28 ± 0.97 µg, respectively (Table 6).

**Figure 8:**
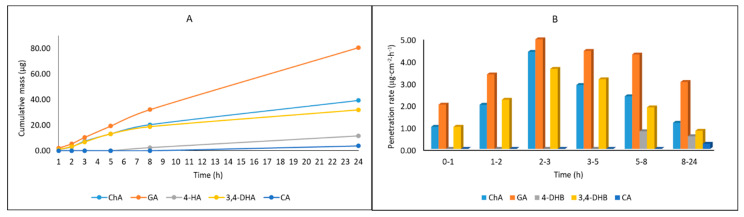
Cumulative mass of phenolic acids in the acceptor fluid during the 24-h penetration (**A**) and the penetration rate (**B**) of phenolic acids through the skin during the 24-h experiment, n = 6.

The cumulative mass of phenolic acids in acceptor fluid and the penetration rate determined at each time interval are presented in Figure 8A,B, respectively. The highest penetration rate to the acceptor fluid was observed in samples collected between 2 and 5 h for GA, ChA, and 3,4-DHB.

**Figure 9:**
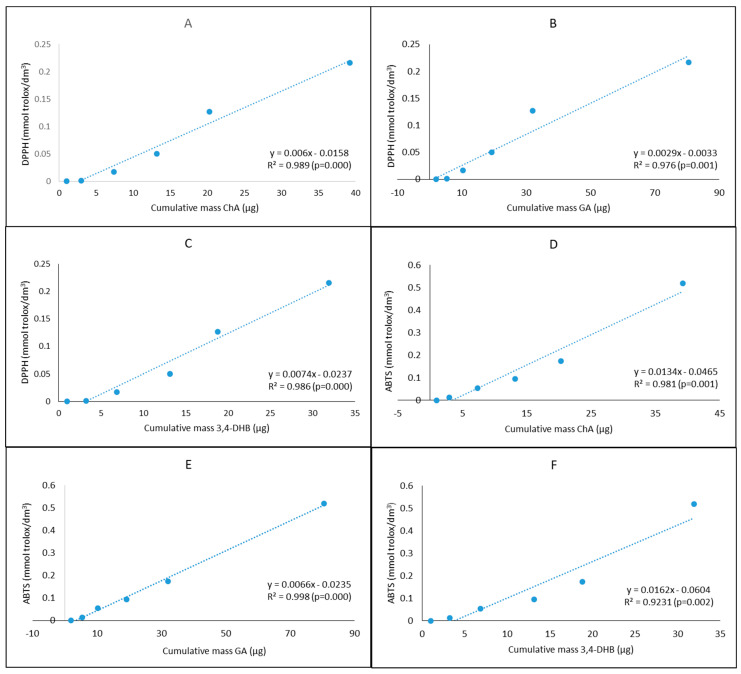
Correlations between the cumulative mass of phenolic acids ChA, GA, 3,4-DHB and the antioxidant activity (DPPH, ABTS) of the acceptor fluid collected during the 24-h permeation study: (**A**) DPPH vs. ChA mass, (**B**) DPPH vs. GA mass, (**C**) DPPH vs. 3,4-DHB mass, (**D**) ABTS vs. ChA mass, (**E**) ABTS vs. GA mass and (**F**) ABTS vs. 3,4-DHB mass. The correlations are presented only for phenolic acids penetrated to the highest degree.

Figure 9 shows the Pearson correlation of the antioxidant activity versus the amount of selected phenolic acids during a 24-h study (Figure 9). A high statistically significant relationship between these parameters was demonstrated; the correlation coefficient ranged from r = 0.923 to r = 0.998.

## 3. Discussion

In recent years, plant extracts containing antioxidants have been used as new alternatives in the production of cosmetics and pharmaceutics with antioxidant and antibacterial properties [21]. *E. angustifolium* has been used for a long time in folk medicine as a useful herb for skin infections, septic wounds, and against important human skin pathogens [1,22]. In addition, due to the high content of polyphenols, including phenolic acids, high antioxidant activity was also observed [3,23,24,25,26,27,28]. We demonstrated that the FEE has antibacterial and antioxidant activity. Simultaneously, some phenolic acids contained in extracts penetrate to and through the skin and accumulate in it, leading, among others, to an antioxidant effect. In our study, to obtain extracts in 70% ethanol, leaves of *E. angustifolium* were harvested in July during the plant flowering phase. Other studies confirmed the high antioxidant activity and the high content of active substances during this phase of vegetation [4,23,29,30,31,32,33].

### 3.1. Chemical Characterization of the FEE and Its Antioxidant Capacity

The FEE analysis by GC-MS showed the content of several groups of compounds, including oxygen derivatives of monoterpene hydrocarbons, unsaturated aliphatic alcohols, camphene derivatives, monocyclic unsaturated terpene ketones, oxygen derivatives of sesquiterpene hydrocarbons, vitamin D derivative, cyclic ether, methyl esters of fatty acids, and methyl ester of oleic acid. Other compounds, also found by Kaškonienė et al. [29], in the extract were β-linalool and eucalyptol. The presence of caryophyllenes (i.e., α- and β-caryophyllene, caryophyllene oxide) was also confirmed in the extracts of dried and fresh leaves of *E. angustifolium* [29] and in essential oils from *E. angustifolium* [34] and *E. hirsutum* [35]. The content of methyl esters of fatty acids, i.e., methyl palmitate and methyl linoleate [34,35], ethyl esters of fatty acids, i.e., ethyl palmitate and ethyl linoleate, and fatty acids, i.e., linoleic acid and oleic acid [34], was also confirmed. Seventeen major chemical components were identified by GC-MS in ethanol extracts of *E. montanum* by Canli et al., wherein a large group of identified compounds was fatty acids (palmitic acid and (Z,Z,Z) 9,12,15-octadecatrienoic acid). Other significant compounds of the extract observed by these authors were: γ-sitosterol, 1-heptacosanol, and 1,2,3-benzenetriol [36]. Several compounds belonging to the terpenes group, i.e., camphor and α- and β-caryophyllene oxide, were found in our study; they were also observed in *E. hirsutum* and *E. angustifolium* by others [35,37]. These compounds are characterized by a strong antibacterial effect [38]. For example, caryophyllene is a natural bicyclic sesquiterpene usually found in various essential oils. It can act as an antimicrobial agent against such pathogens as *P. aeruginosa* and *B. subtilis* [19]. Other compounds of fireweed, also observed in our research, such as linalool and eucalyptol, have strong antibacterial properties as well [19,39,40].

The content of volatile compounds in the plant raw material is primarily affected by the geographical origin, plant chemotype, methods of obtaining the extract, and the solvent used in the extraction. Air drying is the most popular method used to prepare, preserve, and store plant materials for extended periods [29]. Such a preparation method can reduce some compound content; however, it may sometimes have a beneficial effect. Slow drying of *E. angustifolium* herb at ambient temperature and in the dark could increase α- and β-caryophyllene and could form new terpenes: *trans*- and *cis*-anetone, menthol, and aldehydes [29].

In our study, phenolic acids such as ChA, GA, 4-HB, 3,4-DHB, and CA were identified by HPLC, and GA was found in a considerable amount. GA and ChA were also found in the leaves of *E. angustifolium* by Ruszová et al. and Lasinskas et al. [3,41]. Shikov et al. found a higher GA content than other acids identified by authors, including 3,4-DHB [27]. The phenolic acids have been also identified in other varieties of *Epilobium*. Remmel et al. identified many GA in *E. hirsutum* [42]. In contrast, Cando et al. found a low content of hydroxybenzoic and hydroxycinnamic acids and GA in this variety [28]. On the other hand, the higher content of CA and 4-HB in *E. hirsutum* was found by Wojdyło et al. [43]. The observed differences in phenolic acid content may be partly due to different growing conditions, environmental factors, state of ripening, and processing techniques [3,28].

Our research also demonstrated the antioxidant activity of the FEE, which was confirmed by other authors [3,4,24,29,32,44,45,46]. Polyphenols are essential compounds in plants with antioxidant capacity [7,44,47] and antibacterial activity [7,8,48]. In our study, FEE was characterized by a high total polyphenol content; this observation was confirmed by Lasinskas et al. and Shikov et al. [3,27]. Moreover, the results of studies on other *Epilobium* varieties, among others *E. parviflorum*, *E. hirsutum*, *E. adenocaulon*, *E. montanum*, and *E. palustre,* led to a similar conclusion [28,42,43].

### 3.2. Microbiological Assay

The skin and the underlying soft tissue infections are among the most common bacterial infections [10], and Gram-positive as well as Gram-negative bacteria are the main etiological factors [49]. *Streptococcus* spp. are frequently occurring strains classified as Gram-positive, while *P. aeruginosa* is among the Gram-negative strains [10]. In our study, a higher antibacterial activity of the FEE against bacteria of genus *Serratia* and bacteria of genus *Bacillus* than against *Enterococcus*, *Streptococcus*, and *Pseudomonas* genera was found. Battinelii et al. and Kosalec et al. confirmed the antibacterial activity of ethanol extracts of *E. angustifolium* against *B. subtilis*, *E. faecalis*, and *P. aeruginosa* strains [5,31]. Kosalec et al. pointed to a greater sensitivity of *B. subtilis* compared to *P. aeruginosa*, which was confirmed in our study.

According to Bartfay et al., higher antibacterial activity of *E. angustifolium* extracts against *S. aureus*, *E. coli*, and *P. aeruginosa* as compared to antibiotics was observed [50].

Moreover, methanol extracts from seeds of *E. angustifolium*, *E. coloratum*, and *E. glandulosum* showed antibacterial activity against *S. aureus*, *Enterobacter aerogenes*, *Shigella flexneri*, and *P. aeruginosa* [51]. Nicu et al. showed the antibacterial activity of *E. hirsutum* ethanol extracts against *S. aureus*, *S. epidermidis*, *E. coli,* and *P. aeruginosa* strains [52]. The sensitivity of *S. pneumoniae*, *S. pyogenes*, and *S. aureus* strains was also observed with honey obtained from *E. angustifolium* [53].

### 3.3. Skin Penetration

We demonstrated the antioxidant activity in three compartments: (1) plant extract applied to the skin, (2) acceptor fluid after 24 h of penetration, and (3) fluid obtained after skin extraction, collected after the completion of penetration. The antioxidant activity of the fluid obtained after skin extraction was higher than that of the acceptor fluid and indicated the accumulation of ingredients responsible for the antioxidant effect. Alonso et al. demonstrated the high antioxidant activity of methanol porcine skin extract evaluated by the DPPH test after applying compounds with a high antioxidant potential (rutin, quercetin, and trolox). Results of their study suggested a high accumulation of some antioxidants in the skin [54]. In our study, a high accumulation of phenolic acids was also observed. The skin penetration of plant extracts plays an important role. However, the plant active substances can penetrate to a varying degree to tissues, and this parameter depends on their physicochemical properties. To improve the antioxidant properties of cosmetics and/or pharmaceutics, the application of proper original plant substances seems to be essential.

The suitable substances for antioxidant activity enhancement could be plant antioxidants. The topical application of such substances could be helpful to improve the endogenous cutaneous protection system [54]. Evaluation of permeation through the skin is an essential factor to elaborate preparations for the topical delivery of bioactive compounds [55]. The herbal extracts contain a lot of valuable antioxidants, which can accumulate in the skin or penetrate into deeper layers and systemic circulation [9]. The antioxidant effect of plant extracts applied topically is also essential, as oxidative stress can increase the infection severity and could disturb wound healing [16].

In our study, GA, 3,4-DHB, and ChA penetrated to a high degree. The low penetration of CA through the skin was confirmed by Bertges et al., who analyzed the release of phenolic acids from a hydrogel containing 5% coffee seed extract [9]. Marti-Mesters et al. showed penetration of both CA and ChA (applied as pure compounds) through the pig skin [39]. As previously mentioned, the penetration of active substances through the skin also depends on the physicochemical properties, in particular molecular weight and lipophilicity of the compounds [56,57,58]. Higher lipophilicity increases whereas higher molecular weight decreases percutaneous absorption [54].

The vehicle used can have a significant effect on the penetration of active substances through the skin [9,59,60]. In our study, the extracts of *E. angustifolium* in 70% ethanol were applied as a donor solution because ethanol was used in previous studies to prepare an *E. angustifolium* extract and to evaluate antibacterial [31] and antioxidant properties [4]. This concentration of ethanol seems to be optimal for the topical application of the drug [35,61,62,63].

Ethanol is a promoter of transepidermal transport, which affects the effectiveness of active substance penetration into the skin. Ethyl alcohol can reversibly transform the structure of the laminar system of the lipid matrix of the epidermis. As a result, it can facilitate or accelerate the diffusion of particles in the stratum corneum. In addition, ethanol can disrupt the skin barrier’s function by affecting the cells between the cellular cement. It results in loosening the lipid layer and increasing its fluidity and, as a consequence, increases the diffusion of active compounds [64]. The 70% (*v*/*v*) ethanol used in our study could increase the penetration of some phenolic acids. Tuntiyasawasdikul et al. confirmed that the application of ethanol/water mixture increased the penetration of diarylheptanoids from a *Curcuma longa* L. extract as compared to propylene glycol/water solution [62]. Bertges et al. found no CA penetration from the coffee extract in oil-in-water (O/W) emulsion. These authors suggested that this form of vehicle was not suitable for delivering this group of bioactive compounds to the skin [9].

In contrast, Boelzinger et al. showed greater penetration of ChA from microemulsion than from the gel or emulsion [65]. However, an increase of CA penetration was observed through the pig ear after using liposomes [66] and nanostructured lipid carriers [67]. The same substances in different vehicles may penetrate deeper or accumulate in greater amounts in the skin. The lower penetration of antioxidant ingredients through the skin enhances the antioxidant capacity of the stratum corneum. However, increased percutaneous penetration is required if compounds are included in transdermal formulations [55]. In our study, some phenolic acids (CA and 4-HB) penetrated to a low degree. Bertges et al. suggested that in the case of cosmetic preparation, lower penetration to the deeper layers will result in a more significant antioxidant effect in the skin [9].

The polyphenols content in plants correlated with their antioxidant activity [68,69,70]. A significant correlation was demonstrated in our study between the skin penetration of selected phenolic acids and antioxidant activity of the acceptor fluid collected during the 24-h study. It is evident that the total amount of antioxidants in the plant extracts is responsible for the antioxidant activity, and phenolic acids seem to play an essential role [30,71].

In conclusion, this study confirmed that fireweed ethanol-water extracts (FEEs) contain a lot of active substances and show antioxidant and antibacterial activity. In our study, the penetration of selected phenolic acids included in the FEE through the human skin was observed. The obtained results indicate the possibility to use the FEE as an ingredient, for example, in cosmetics and pharmaceutics applied to the skin. Fireweed ethanol extract may be a promising alternative to “synthetic” preparations with antioxidative and antibacterial properties.

## 4. Materials and Methods

### 4.1. Chemicals

2,2-diphenyl-1-picrylhydrazyl (DPPH), 6-hydroxy-2,5,7,8-tetramethylchroman-2-carboxylic acid (trolox), 2,2′-azino-bis(3-ethylbenzothiazoline-6-sulfonic acid) (ABTS), 2,4,6-tripyridyl-s-triazine (TPTZ), 3,4-dihydroxybenzoic acid, chlorogenic acid, and caffeic acid were purchased from Sigma Aldrich (Poznań, Poland); Folin–Ciocalteu reagent, gallic acid, 4 hydroxybenzoic acid, disodium phosphate, and potassium dihydrogen phosphate from Merck, Darmstadt (Germany); sodium acetate anhydrous, potassium persulfate, potassium acetate, 99.5% acetic acid, 36% hydrochloric acid, sodium chloride, potassium chloride, ethanol, and methanol were from Chempur (Piekary Śląskie, Poland), whereas acetonitrile for HPLC was from J.T. Baker (the Netherlands). All reagents were of analytical grade.

### 4.2. Plant Material

The plant material was collected during the flowering phase in July in Poland (N 53°23′18″, E 14°28′56″) from the natural state. The plants were selected randomly from different, near located places. Five samples were harvested and combined into one collective sample. The aerial part of *E. angustifolium* herb was harvested during the massive blooming period [23,29]. The plant material was dried at room temperature in a well-ventilated area to a constant weight [29]. Samples were deposited in the plant material storage room (No. EEA-AM2019-03) at the Chair and Department of Cosmetic and Pharmaceutical Chemistry of the Pomeranian Medical University. The plant material was ground in the grinder and sieved using a circular-hole screen (8 mm mesh). Five grams of dried raw material were extracted with 100 cm^3^ 70% (*v*/*v*) ethanol [18] for 30 min in an ultrasonic bath at a frequency of 40 kHz. Extracts were filtered through a Whatman paper filter (codified EEA03) and thereafter stored at +4 °C until analyses. The extracts were applied to in vitro skin penetration studies. The obtained samples and initial extracts were evaluated using HPLC and GC-MS methods, and microbiological and antioxidant activity was also determined.

### 4.3. GC-MS and HPLC Analysis

The qualitative and quantitative composition of the FEE was evaluated by gas chromatography-mass spectrometry (GC-MS). Chromatographic analyses were performed with TRACE GC series apparatus equipped with a VOYAGER mass detector using a DB5 capillary column (30 m × 0.25 µm × 0.5 µm). The following separation parameters were used for the analysis: helium flow of 1.0 cm^3^/min, sample chamber temperature of 240 °C, and detector voltage of 350 V. The thermostat temperature increased according to the following program: isothermal at 50 °C for 1 min, increased at 8 °C/min, isothermal at 260 °C for 5 min, and then cooled to 50 °C. The sample partition coefficient in the dispenser was 20, the volume of the dispensed sample was 1 mm^3^, and the ion mass range was 25–350 mV/z. The quantitative composition of individual compounds was determined, assuming that the sum of all identified compounds is 100%.

The concentration of test compounds in the FEE was determined by high-performance liquid chromatography (HPLC-UV), using the HPLC system from Knauer (Berlin, Germany). The tested components were separated on a 125 mm × 4 mm column containing Hyperisil ODS, particle size 5 µm. The mobile phase consisted of acetonitrile, 1% acetic acid, and MeOH (45:45:10 by vol.), the flow rate was 1 cm^3^/min. Twenty cubic millimeters of the sample were injected onto the column. The correlation coefficient of the calibration curve was 0.9964 (y = 277926x + 0.226, t_R_-2,286 min) for gallic acid, 0.9992 for chlorogenic acid (y = 53905x + 9.831, t_R_-5,639 min), 0.999 for 4-hydroxybenzoic acid (y = 26889x + 3.5605, t_R_-4,305 min), 0,999 for 3,4-dihydroxybenzoic acid (y = 78007x − 1.1925, t_R_-2,953 min), and 0.9994 for caffeic acid (y = 67950x + 5.141, tR-6,023). The extracts were 12-fold diluted before injection. All samples were analyzed three times.

### 4.4. Evaluation of the Antioxidant Capacity Using DPPH, ABTS, and Folin–Ciocalteu Methods

Antioxidant activity and total polyphenol content in plant extracts applied to the skin, in acceptor fluid collected after 24-h penetration, and in the fluid obtained after skin extraction following penetration completion were evaluated.

The scavenging activity of DPPH stable free radicals was measured as described previously [68,72,73]. Shortly, an aliquot of 0.15 cm^3^ of the studied samples was mixed with 2.85 cm^3^ of 0.3 mM DPPH radical solution dissolved in 96% (*v*/*v*) ethanol. The absorbance at 517 nm of the DPPH working solution was adjusted to 1.00 ± 0.02 with 70% (*v*/*v*) ethanol. After 10 min of incubation in the dark at room temperature, measurement of absorbance at 517 nm against 70% (*v*/*v*) ethanol was performed using Hitachi UV-Vis Spectrophotometer U-5100. Three independent samples of each examined extract were prepared. As a reference, 6-hydroxy-2,5,7,8-tetramethylchroman-2-carboxylic acid (trolox) was applied. The results are presented as trolox equivalents (TEAC) in mmol trolox/dm^3^.

The procedure applied to evaluate ABTS radical scavenging activity was described previously [72]. Shortly, 7 mM solution of ABTS (2,2′-azino-bis(3-ethylbenzothiazoline-6-sulfonic acid)) in a 2.45 mM aqueous solution of potassium persulfate was used as a stock solution. After dissolving the components, the solution was incubated for 24 h, in the dark at room temperature, then diluted with 50% (*v*/*v*) methanol to obtain a working solution of absorbance of 1.00 ± 0.02 at 734 nm. The antioxidant activity was measured as follows: 2.5 cm^3^ of working ABTS solution and 0.025 cm^3^ of a studied sample were introduced into the spectrophotometric cuvette. After 6 min of incubation at room temperature, absorbance at 734 nm was measured. Each extract was evaluated in triplicate. As previously, the results were expressed as trolox equivalents (TEAC) in mmol trolox/dm^3^.

Total polyphenol content was determined with the Folin–Ciocalteu method as described previously [4]. Shortly, to 0.15 cm^3^ of the studied sample, 0.15 cm^3^ of tenfold diluted Folin–Ciocalteu reagent, 1.35 cm^3^ of 0.01 M sodium carbonate solution, and 1.35 cm^3^ of water were added and mixed. The cuvette was sealed with a stopper and then incubated for 15 min at room temperature. After this time, the spectrophotometric measurement was carried out at 765 nm. As previously, three samples were prepared for each extract. Gallic acid (GA) was applied as a standard, and results were expressed as gallic acid equivalents (GAEs) in mmol GA/dm^3^.

### 4.5. Microbiological Analysis

The microbiological analysis included the effect of the FEE on ten bacterial strains. The following strains of microorganisms were used in the studies: *S. lutea* ATCC 9341, *S. marcescens*, *E. faecalis* ATCC 29212, *E. faecium*, *S. pneumoniae* ATCC 49619, *P. aeruginosa* ATCC 2753, *P. fluorescens*, *B. subtilis*, *B. pseudomycoides*, and *B. thuringiensis.* The test microorganisms’ sensitivity to the tested extract was determined by the agar medium’s diffusion method using the well variant [74,75]. For bacterial cultivation, TSA (tryptic-soya agar) medium was used. The appropriate medium (20 cm^3^) was poured into Petri plates with a diameter of 90 mm. After solidifying the medium, five wells with a diameter of 4 mm were bored out using a sterile cork borer. On such prepared Petri dishes, 0.1 cm^3^ of a 24-h bacterial culture in a liquid tryptone-soybean (TSB) medium with 0.25% Tween 20 was introduced. The inoculum was spread evenly over the surface of the medium using a glass spatula. The inoculated plates were allowed to absorb the liquid inoculum for about 60 min altogether. Next, 10 mm^3^ of FEE solution with a concentration of 12.5%, 25%, 50%, and 100% (without dilution) were introduced into the four wells. Each Petri dish well contained 1.25 mg, 2.5 mg, 5.0 mg, and 10 mg of the undiluted extract, respectively. As a control, 10 mm^3^ of 70% ethanol was placed in the well in the dish’s center. The Petri plates were incubated at 37 °C for 72 h, and after that, the zones of inhibition were measured using a meter ruler. The inhibitory effect of test extract was assessed based on the zone of complete inhibition of the cultured strain growth. Measurements were made every 24 h, and as a result, the score after 72 h was used.

### 4.6. In Vitro Skin Permeation Studies of the FEE

The permeation experiments were performed in the Franz diffusion cells (SES GmbH Analyse Systeme, Bechenheim, Germany) with a diffusion area of 1 cm^2^. The donor chamber volume was 2 cm^3^, and the volume of the acceptor chamber was 8 cm^3^. The acceptor chamber was filled with PBS solution (pH 7.4). In each diffusion unit, a constant temperature of 32.0 ± 0.5 °C [9] was maintained via a thermostat (VEB MLW Prüfgeräte-Werk type 3280, Leipzig, Germany). The acceptor chamber content was stirred with a magnetic stirring bar at the same speed for all cells. Human abdominal skin obtained after plastic surgery was used. Each volunteer gave written informed consent, and the study was approved by the Ethical Committee of Pomeranian Medical University in Szczecin (KB-0012/02/18). The skin of 0.5 mm in thickness was dermatomed. The skin was then divided into 2 cm × 2 cm pieces. The skin samples were wrapped in aluminum foil and stored in a freezer at −20 °C until use, not longer than three months. This frozen storage time was safe to keep skin barrier properties [76]. On the day of the experiment, the skin samples were slowly thawed at room temperature for 30 min and were hydrated by PBS pH 7.4 [77,78,79]. Undamaged pieces of skin (checked by measuring skin impedance) were placed in the Franz diffusion cell between donor and acceptor chamber. After placing the skin in the Franz diffusion cells, all chambers were allowed to equilibrate at 37 °C for 15 min. The measurement of skin impedance checked its integrity. For this purpose, an LCR meter 4080 (Voltcraft LCR 4080, Conrad Electronic, Germany), operated in parallel mode at an alternating frequency of 120 Hz (error at kΩ values <0.5%), was used. The tips of measuring probes were immersed in the donor and acceptor chamber, filled with PBS (pH 7.4) as described previously [80,81]. Only skin samples with impedance >3 kΩ were used. These values are similar to the electrical resistance of human skin [82]. Thereafter, a defined dose (0.5 cm^3^) of the test extract was applied to the skin’s outer side. All donor chambers were closed with plastic stoppers to prevent the evaporation of the solution.

The penetration study was carried for 24 h. At the time points of 1, 2, 3, 5, 8, and 24 h, 0.8 cm^3^ of acceptor samples were withdrawn and the chamber was refilled with the same volume of a fresh buffer of the same pH. The phenolic acid concentrations in the acceptor phase were measured by the HPLC method. The cumulative mass (µg) of each phenolic acid studied was calculated based on the obtained concentration. The antioxidant activity of the samples collected after completing the penetration study was also tested. After 24 h of the experiment, the diffusion cells were disassembled, and the skin samples were analyzed for the content of selected phenolic acids and their antioxidant activity.

The accumulation of the tested compounds in the skin after penetration and antioxidant activity of this skin were determined using a modification of the methods described by Janus et al., Alonso et al., Haq and Michniak-Kohl, and Rubio et al. [54,55,56,77,83]. The procedure was as follows. After 24 h of the experiment, each skin sample was removed and carefully rinsed in PBS (pH 7.4) [81]. The skin was then cut around the diffusion area (1 cm^2^) and dried at room temperature. Each of 1 cm^2^ skin samples was cut into small pieces, placed in 2 cm^3^ methanol, and incubated for 24 h at 4 °C. After this time, skin samples were homogenized for 3 min using a homogenizer (IKA^®^T18 digital ULTRA TURRAX, Germany). The homogenate was centrifuged at 3500 rpm for 5 min. The supernatant was collected for subsequent HPLC and spectrophotometric analyses with pure methanol applied as a control. Before injection onto the HPLC column, the collected samples were diluted threefold. Accumulation of the phenolic acids in the skin was calculated by dividing the amount of the substances remaining in the skin by mass of skin sample and was expressed as the mass of phenolic acid per mass of the skin (µg/g). The antioxidant activity of the solution obtained after skin extraction was also determined.

### 4.7. Statistical Analysis

Results are presented as the mean ± standard deviation (SD). The Pearson test was used to demonstrate the correlation between the penetration of selected phenolic acids and their antioxidant activity. With microbiological analysis, a one-way analysis of variance was used (ANOVA). The significance of differences between individual groups was evaluated with Tukey’s test (α < 0.05). A cluster analysis was carried out to determine the characteristics of the extract action on the tested bacteria. On this basis, groups of bacteria with a similar reaction of extracts were determined, as well as the effect of different doses of the extract on the bacteria. Statistical calculations were done using Statistica 13 PL software (StatSoft, Polska).

## Figures and Tables

**Table 1:** Major components of the fireweed ethanol-water extract (FEE) determined with gas chromatography-mass spectrometry (GC-MS).

No	Retention Time	Compound Name	Area (%)
1	9.68	Eucalyptol	10.3
2	11.33	β-Linalool	14.8
3	11.57	Camphor	0.9
4	12.21	α-Terpineol	0.7
5	14.46	α-Terpinyl acetate	1.1
6	17.74	α-Caryophyllene oxide	1.2
7	18.39	β-Caryophyllene oxide	1.2
8	19.41	24,25-Dihydroxycholecalciferol	7.5
9	20.41	5-Hexadecyloxy-2-pentadecyl-1,3-dioxane	5.2
10	21.10	Methyl palmitate	15.2
11	22.80	Methyl linoleate	9.6
12	22.86	Methyl oleate	32.2

**Table 2:** Concentrations of phenolic acids of the FEE. Mean (±standard deviation), n = 6.

Phenolic Acid Mg/Dm^3^				
ChA	GA	4-HB	3,4-DHB	CA
64.35 ± 0.53	241.36 ± 4.42	118.16 ± 4.49	165.19 ± 5.59	54.29 ± 2.25

ChA: chlorogenic acid; GA: gallic acid; CA: caffeic acid; 4-HB: 4-hydroxybenzoic acid; 3,4-DHB: 3,4-dihydroxybenzoic acid.

**Table 3:** Antioxidant activity of the FEE. Mean (±standard deviation), n = 6.

Total Polyphenols Mmol GA/Dm^3^	DPPH Mmol Trolox/Dm^3^	ABTS Mmol Trolox/Dm^3^
1.94 ± 0.06	3.68 ± 0.02	12.98 ± 0.04

**Table 4:** Inhibitory zones (mm) of the tested strains after applying the FEE at different concentrations. Results were from three independent experiments (n = 3). Mean (±standard deviation).

Strain	Extract Concentration			
	100%	50%	25%	12.50%
*Serratia lutea*	16.00 ± 0.32 a	15.00 ± 0.06 a	13.50 ± 0.50 ab	8.00 ± 2.00 c
*Serratia marcescens*	15.00 ± 0.6 a	13.50 ± 0.05 a	10.00 ± 0.06 b	7.00 ± 1.00 c
*Enterococcus faecalis*	7.00 ± 0.12 a	6.00 ± 0.06 b	5.00 ± 0.06 c	5.00 ± 0.06 c
*Enterococcus faecium*	7.00 ± 0.01 a	6.00 ± 0.01 b	5.00 ± 0.05 c	5.00 ± 0.06 c
*Streptococcus pneumoniae*	7.00 ± 0.01 a	6.00 ± 0.06 b	5.00 ± 0.15 c	5.00 ± 0.06 c
*Pseudomonas aeruginosa*	6.00 ± 0.06 a	5.00 ± 0.06 b	4.00 ± 0.06 c	4.00 ± 0.010 c
*Pseudomonas fluorescens*	6.00 ± 0.12 a	6.00 ± 0.10 a	6.00 ± 0.06 a	6.00 ± 0.06 a
*Bacillus subtilis*	11.00 ± 0,80 a	9.50 ± 1.15 b	7.00 ± 1.04 c	6.50 ± 0.55 c
*Bacillus pseudomycoides*	11.50 ± 0.58 a	9.00 ± 1.00 b	7.50 ± 0.50 c	6.00 ± 0.06 c
*Bacillus thuringiensis*	9.00 ± 0.52 a	7.50 ± 0.58 b	6.00 ± 0.06 c	5.50 ± 0.50 c

Different letters: values differ significantly between the analyzed concentrations.

**Table 5:** Mean (±standard deviation) FEE antioxidant activity of the extract applied to the skin, solution obtained after skin extraction and acceptor fluid collected after 24-h penetration (n = 6).

	DPPH Mmol Trolox/Dm^3^	ABTS Mmol Trolox/Dm^3^	Folin-Ciocalteu Mmol GA/Dm^3^
extract applied to the skin	3.683 ± 0.048	12.985 ± 0.045	1.941 ± 0.010
extract after skin extraction following 24-h penetration	0.456 ± 0.034	1.622 ± 0.57	1.114 ± 0.106
acceptor fluid after 24-h penetration	0.216 ± 0.078	0.519 ± 0.107	0.591 ± 0.148

**Table 6:** The content of phenolic acids in acceptor fluid and extract obtained after the 24-h penetration study.

		ChA	GA	4-HB	3,4-DHB	CA
cumulating in the skin	µg/g skin	110.46 ± 7.60	335.54 ± 51.50	176.18 ± 13.40	266.67 ± 28.43	119.07 ± 20.88
acceptor fluid after 24 h of penetration	µg	30.28 ± 0.97	80.51 ± 8.27	11.57 ± 3.77	31.93 ± 1.116	3.70 ± 0.96

## Data Availability

The data presented in this study are available in this article.

## Sample Availability

Samples of the compounds are available from the authors.

## Abbreviations

FEE	fireweed ethanol-water extracts
DPPH	2,2-diphenyl-1-picrylhydrazyl
ABTS	2,2′-azino-bis(3-ethylbenzothiazoline-6-sulfonic acid)
TPTZ	2,4,6-tripyridyl-s-triazine
GA	gallic acid
ChA	chlorogenic acid
3,4-DHB	3,4-dihydroxybenzoic acid
4-HB	4-hydroxybenzoic acid
CA	caffeic acid
PhA	phenolic acids
GC-MS	gas chromatography coupled with mass spectrometry
HPLC	high-performance liquid chromatography
TSA	tryptic-soya agar
TSB	liquid tryptone-soybean
TEAC	trolox equivalent antioxidant capacity
GAE	gallic acid equivalents
ROS	reactive oxygen species
